# Frame Transmission Efficiency-Based Cross-Layer Congestion Notification Scheme in Wireless Ad Hoc Networks

**DOI:** 10.3390/s17071637

**Published:** 2017-07-15

**Authors:** Huaguang He, Taoshen Li, Luting Feng, Jin Ye

**Affiliations:** 1 School of Electronic and Information Engineering, South China University of Technology, Guangzhou 510641, China; hehuaguang98@gxu.edu.cn; 2 School of Computer and Electronic Information, Guangxi University, Nanning 530004, China; tshli@gxu.edu.cn (T.L.); fltinger@foxmail.com (L.F.); 3 Guangxi Key Laboratory of Multimedia Communications and Network Technology (Cultivating Base), Guangxi University, Nanning 530004, China

**Keywords:** ad hoc networks, frame transmission efficiency, explicit congestion notification

## Abstract

Different from the traditional wired network, the fundamental cause of transmission congestion in wireless ad hoc networks is medium contention. How to utilize the congestion state from the MAC (Media Access Control) layer to adjust the transmission rate is core work for transport protocol design. However, recent works have shown that the existing cross-layer congestion detection solutions are too complex to be deployed or not able to characterize the congestion accurately. We first propose a new congestion metric called frame transmission efficiency (i.e., the ratio of successful transmission delay to the frame service delay), which describes the medium contention in a fast and accurate manner. We further present the design and implementation of RECN (ECN and the ratio of successful transmission delay to the frame service delay in the MAC layer, namely, the frame transmission efficiency), a general supporting scheme that adjusts the transport sending rate through a standard ECN (Explicit Congestion Notification) signaling method. Our method can be deployed on commodity switches with small firmware updates, while making no modification on end hosts. We integrate RECN transparently (i.e., without modification) with TCP on NS2 simulation. The experimental results show that RECN remarkably improves network goodput across multiple concurrent TCP flows.

## 1. Introduction

Wireless ad hoc networks are widely used in military and civil mobile communication, where fixed communications infrastructures (i.e., base station) are not available. Different from WLAN (Wireless Local Area Network), the ad hoc network is a multi-hop wireless network without a centralized coordinator. Each node works as a router to forward the packets from other nodes. In this special wireless network, TCP (Transmission Control Protocol) flows traverse multiple hops before reaching the destination node or sink node. To provide reliable communications in wireless ad hoc networks, it is critical to design an efficient TCP congestion control mechanism [1].

However, the performance of the wireless ad hoc network will be degraded if the traditional TCP protocol is utilized. The reason is that TCP congestion control has an implicit assumption that any packet loss is due to the buffer overflow. In fact, as long as the buffer size at each wireless node is reasonably large, most packet losses are due to wireless channel contention, namely, MAC (Media Access Control) layer competition [2,3,4].

Therefore, several cross-layer schemes are proposed to alleviate congestion in the wireless channel [5,6,7,8]. For example, a cross-layer ECN (Explicit Congestion Notification) scheme was proposed to perceive the link congestion according to the retransmission counter at the MAC layer. In this scheme, the retransmission counter at the MAC layer is used as the congestion metric to trigger the ECN mechanism. ECN is the explicit congestion notification mechanism of the IP layer. When network congestion occurs, the sender can adjust the congestion window and reduce the sending rate by the ECN mark from the receiver. Differently from [9], Yue Peng et al. utilized the frame service delay at the MAC layer as the congestion metric to mark or drop packets [10,11,12]. WCCP (Wireless Congestion Control Protocol) was proposed to dynamically adjust the congestion window of TCP according to the channel business ratio, which presents the extent of channel congestion in a more accurate way [13]. These cross-layer methods improve the efficiency and fairness of wireless ad hoc networks [14,15]. However, the retransmission counter and the frame service delay are not precise enough to detect congestion from the MAC layer, while WCCP is too complex to implement in existing transport protocols.

Therefore, this paper proposes an MAC congestion metric called frame transmission efficiency, describing channel congestion in a more precise manner. Furthermore, combining this congestion metric with ECN, we propose a cross-layer ECN mechanism, RECN (ECN and the ratio of successful transmission delay to the frame service delay in the MAC layer, namely, the frame transmission efficiency), which is based on frame transmission efficiency and adjusts the transport sending rate through a standard ECN signaling method, without any modification to transport protocols.

The remainder of this paper is structured as follows. Section 2 describes several congestion metrics at the MAC layer and proposes a congestion notification scheme RECN. Section 3 gives the definition and analysis of the frame transmission efficiency to demonstrate the relationship between the frame transmission efficiency and the link layer congestion. Section 4 gives the protocol design of RECN. In Section 5, the simulation results show that our proposed congestion notification mechanism significantly outperforms traditional TCP in wireless multi-hop ad hoc networks. Section 6 is our conclusion.

## 2. Wireless Channel Congestion Detection

How to detect wireless channel congestion is important for congestion control design in wireless networks. Recent works have proposed several congestion metrics at the MAC layer.

(1) Retransmission counter: There exists a correlation between the number of RTS (Request to Send) retransmissions and channel congestion extent at the MAC layer [16,17]. In [4], when the number of RTS retransmissions is greater than two, the wireless channel will be deemed as congested, and the TCP sender will decrease its sending rate. Though this method is very easy to deploy, the number of RTS retransmissions is not able to describe the congestion state accurately.
(2) Frame Service Delay (FSD): The frame service delay is the interval from the time that the MAC layer begins to sense the channel for data transmission to the time that the acknowledgment is received successfully, which includes collision time and transmission time [18]. The greater the service delay is, the higher the probability of network congestion is. However, the hops that the flows are traveling through and the frame size greatly affect the service delay. Subsequently, the threshold value of the service delay is difficult to determine, resulting in the inaccuracy of congestion estimation.
(3) Channel business ratio: The channel business ratio is defined as the ratio of link layer busy time (including collision time and data successful transmission time) to the total time [19,20,21,22]. It can be shown in Equation (1).

(1) $$R_{b}=1-\frac{p_{i}\sigma }{p_{i}\sigma +p_{s}T_{suc}+p_{c}T_{col}}$$

where σ is the length of the backoff time slot, $p_{i}$ is the probability that the observed backoff time slot is idle, $p_{s}$ is the probability that there is one successful transmission, $p_{c}$ is the collision probability that there are at least two concurrent transmission at the same time slot, $T_{suc}$ is the average time period associated with one successful transmission and $T_{col}$ is the average time period associated with collisions.

As shown in Equation (1), the channel business ratio accurately reflects the contention and collision at the MAC layer. When the channel business ratio $R_{b}$ increases, the channel utilization and network throughput will become higher. However, the channel business ratio is too complex to be deployed on the TCP protocol. Specifically, the key problem lies in the time interval involved in calculating $R_{b}$ being difficult to set. The ideal situation appears only when the time interval is exactly equal to the transmission cycle of a frame. However, the transmission cycle of a frame will change with many factors, such as frame length, flow concurrency and channel status.

Compared with the above solutions, this paper proposes a congestion notification scheme RECN, which uses the frame transmission efficiency as the congestion metric. With only a very small modification at the MAC layer, frame transmission efficiency characterizes the channel congestion status more accurately and can be deployed on the TCP protocol using the standard ECN signal method, improving the efficiency and fairness of the resource distribution in wireless ad hoc networks.

## 3. Definition and Theory Analysis

RECN is the cross-layer congestion notification scheme, based on the frame transmission efficiency $R_{FTE}$ and using the standard ECN mechanism. RECN works at the MAC layer and adjusts the sending rate of the transport protocol automatically and transparently. In this scheme, the frame transmission efficiency is the key point, being put as the new congestion metric. Thus, in this section, we give the definition and analysis of the frame transmission efficiency to demonstrate the relationship between the frame transmission efficiency and the link layer congestion.

### 3.1. Definition

IEEE (Institute of Electrical and Electronics Engineers) 802.11 is the standard MAC protocol of wireless networks. It includes RTS/CTS (Request To Send/Clear To Send) and basic access mechanisms for data transmission in DCF mode [23].

In the RTS/CTS scheme, the RTS frame is sent to the reserve channel when the TCP source wants to transmit the data packet. If the receiver successfully receives the RTS frame, it returns a CTS (Clear To Send) frame. After that, the data and the ACK (Acknowledgment) frame will be transmitted respectively. Only the RTS frame may have a collision in this mechanism. Since the RTS frame has no data and is a short frame, it avoids long data frame collision and the hidden terminal problem; while in the basic access scheme, the data will be sent directly once nodes access the channel instead of sending RTS.

In the DCF (Distributed Coordination Function) mode, each node uses the CSMA/CA (Carrier Sense Multiple Access with Collision Avoidance) mechanism to compete for wireless channel. Firstly, the sender will detect the channel. If the idle time lasts for the DIFS (Distributed Inter-frame Spacing) time interval and a random backoff time, RTS or the data frame will be sent. If no CTS or ACK is received within a specified period, this frame is considered lost. Then, the transmitter will double the size of the contention window and choose a new backoff timer.

**Figure 1:**
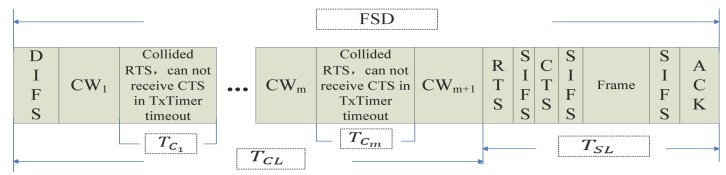
The delay of RTS/CTS (Request To Send/Clear To Send) mechanism.

**Figure 2:**
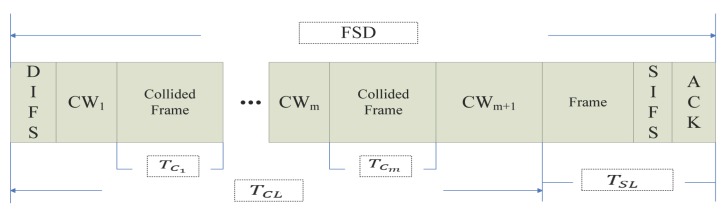
The delay of the basic access mechanism.

The model of MAC layer service delay is shown in Figure 1 and Figure 2. No matter which way it works, we define the frame transmission efficiency $R_{FTE}$ as the ratio of successful transmission delay to the frame service delay in the MAC layer, that is

(2) $$R_{FTE}=\frac{T_{SL}}{T_{FSD}}$$

where $T_{SL}$ is the delay of data successful transmission, which refers to the interval from the start of sending data to the time ACK is received. $T_{FSD}$ is the total frame service delay from listening on the channel for transmitting this frame to this frame being transmitted successfully.

From the above definition, $R_{FTE}$ can reflect the state of channel congestion. When the channel is in a congested state, $R_{FTE}$ is smaller. On the contrary, $R_{FTE}$ is larger when the network communication is in good condition.

### 3.2. Theory Analysis

We derive frame transmission efficiency:

(3) $$R_{FTE}=\frac{T_{SL}}{T_{FSD}}=\frac{E(T_{SL})}{E(T_{FSD})},$$

where $E(T_{SL})$ is the mathematic expectation of $T_{SL}$. $E(T_{FSD})$ is the mathematic expectation of $T_{FSD}$.

(1) The numerical value of $T_{SL}$: As we can see in Figure 1 and Figure 2, the time that a frame is successfully transmitted with the RTS/CTS mechanism is:
  (4) $$T_{SL}=T_{RTS}+T_{CTS}+T_{F}+T_{ACK}+3T_{SIFS},$$
  where $T_{F}$ is the time of the data frame transmission. The time that a frame is successfully transmitted while using the basic access mechanism is:
  (5) $$T_{SL}=T_{F}+T_{SIFS}+T_{ACK}.$$
  According to the system parameters, $T_{SIFS}$ is 10 μs. The lengths of RTS, CTS and ACK are 160 bits, 112 bits and 112 bits, respectively. Thus, $T_{SL}$ is determined by the size of the data frame and is independent of the number of collisions, that is $E(T_{SL})$ = $T_{SL}$. If the size of the data frame is determined, $T_{SL}$ will be a constant value.
(2) The numerical value of $T_{FSD}$: The numerical value of $T_{FSD}$ is:
  (6) $$T_{FSD}=T_{CL}+T_{SL},$$
  where:
  (7) $$T_{CL}=T_{DIFS}+\sum_{j=0}^{N}\left(T_{C_{j}}+T_{CW_{j}}\right)+T_{CW_{N+1}},$$
  where $T_{CL}$ is the total time of congestion. $T_{C_{j}}$ is the time of each congestion. $T_{CW_{j}}$ is the time of congestion waiting. *N* is the number of collisions, and the range of *N* is period [1, *m*], where *m* is the maximum number of collisions.

Next, we will calculate the mathematic expectation of $T_{FSD}$.

(8) $$E\left(T_{FSD}|N=k\right)=E\left(T_{CL}\right)+E\left(T_{SL}\right)=T_{DIFS}+E(\sum_{j=0}^{k}\left(T_{C_{j}}+T_{CW_{j}}\right))+E\left(T_{CW_{k+1}}\right)+E\left(T_{SL}\right),$$

that is

(9) $$E\left(T_{FSD}|N=k\right)=\sum_{k=0}^{m}\left(T_{DIFS}+E\left(\sum_{j=0}^{k}\left(T_{C_{j}}+T_{CW_{j}}\right)\right)+E\left(T_{CW_{k+1}}\right)+E\left(T_{SL}\right)\right)\times P(N=k)$$

where P(N=k) is the function of the collision numbers *k*.

We have $E(T_{SL})=T_{SL}$; thus:

(10) $$E\left(T_{FSD}|N=k\right)=\sum_{k=0}^{m}\left(T_{DIFS}+E\left(\sum_{j=0}^{k}\left(T_{C_{j}}+T_{CW_{j}}\right)\right)+T_{CW_{k+1}}+T_{SL}\right)\times P(N=k)$$

With the RTS/CTS mechanism, there is:

(11) $$T_{C_{j}}=\left\{\begin{array}{cc} 0 & j=0\\ T_{RTS}+T_{SIFS}+T_{CTS} & 0<j\leq m \end{array}\right..$$

With the basic access mechanism, there is:

(12) $$T_{C_{j}}=\left\{\begin{array}{cc} 0 & j=0\\ T_{CF}+T_{SIFS}+T_{ACK} & 0<j\leq m \end{array}\right.,$$

where $T_{CF}$ is the time of collision data frame transmission.

According to the default value of the system parameters, $T_{SIFS}$ is 10 μs. The lengths of RTS, CTS and ACK are 160, 112 and 112 bits, respectively. Thus, $T_{C_{j}}$ is determined by the size of the collision data frame. If the packet size is fixed, there is $T_{F}=T_{CF}$. Thus, $\sum_{j=0}^{k}T_{C_{j}}$ is a constant, named *G*.

According to the IEEE 802.11 standard, the node uses the binary backoff algorithm to reserve the channel and send data. In the binary backoff algorithm, an integer is selected as the backoff time slot number $W_{j}$ randomly from the uniformly-distributed period [0, CW]. The backoff window CW will be exponential increased with the number of retransmissions *j*, that is $CW_{j}=2^{j}\times CW_{min},j\in \left[0,M\right]$, where $CW_{min}$ is the constant and *M* is the maximum backoff stage. When the node performs the backoff algorithm in the first time, CW will be $CW_{min}$. The following backoff time slot will be $CW_{j}=2^{j-1}\times CW_{min}$. When the number of collisions exceeds *M*, there is $W_{j}=2^{M-1}\times CW_{min}$. Hence the average backoff time is:

(13) $$E\left(T_{CW_{j+1}}\right)=\left\{\begin{array}{cc} 2^{j-1}CW_{min}*E\left(T_{slot}\right) & 0<j\leq m\\ 2^{M-1}CW_{min}*E\left(T_{slot}\right) & j>M \end{array}\right.$$

where $E(T_{slot})$ is the equivalent time slot length.

It is presumed that there are *n* saturated sites, and each of them always has packets in the queue and is contending for the channel. The transmission probability of each site is τ. Then, the probability that the MAC layer is in an idle time slot is defined as:

(14) $$p_{i}={\left(1-\tau \right)}^{n}$$

We denote $p_{s}$ as the successful transmission probability. That is to say, there is only one site transmitting data without collision from the other n−1 sites in the current slot. It can be expressed as:

(15) $$p_{s}=C_{n}^{1}\tau {\left(1-\tau \right)}^{n-1}=n\tau {\left(1-\tau \right)}^{n-1}$$

The collision probability $p_{c}$ is:

(16) $$p_{c}=1-p_{i}-p_{s}$$

In a time slot, the channel may be in an idle time slot, or in a collision, or transmitting the data successfully. Hence, there is:

(17) $$E\left(T_{slot}\right)=p_{i}\sigma +p_{s}T_{SL}+p_{c}G$$

where σ is the length of the backoff time slot, and it is a constant.

From the above formulas, $p_{s}$ and $p_{i}$ can be replaced by τ and *n*, and $T_{SL}$ and *G* can be used as constants when the packet size is fixed. Thus, $E(T_{slot})$ is determined by τ, *n* and $p_{c}$.

P(N=n) obeys a geometric distribution [10]. That is:

(18) $$P\left(N=k\right)=\left\{\begin{array}{cc} {p_{c}}^{k}\times \left(1-p_{c}\right) & 0\leq k<m\\ {p_{c}}^{k} & k=m \end{array}\right.$$

Combining Equations (10) and (18), we get:

(19) $$E\left(T_{FSD}\right)=\left\{\begin{array}{ll} \frac{CW_{min}\times E\left(T_{slot}\right)\times \left(1-2^{M}{p_{c}}^{M+1}-...-2^{m-1}{p_{c}}^{m}\right)}{2(1-2p_{c})}\\ +\left(T_{DIFS}+T_{SL}\right)\times \left(1-{p_{c}}^{m}\right)+G\times \frac{p_{c}-{p_{c}}^{m+1}}{1-p_{c}} & m<M\\ \left(T_{DIFS}+T_{SL}\right)\times \left(1-{p_{c}}^{m}\right)+\frac{CW_{min}\times E\left(T_{slot}\right)\times \left(1-2^{m}{p_{c}}^{m}\right)}{2(1-2p_{c})}\\ +G\times \frac{p_{c}-{p_{c}}^{m+1}}{1-p_{c}} & m>M \end{array}\right.$$

As shown in Equation (19), $E(T_{FSD})$ is determined by τ, *n* and $p_{c}$, when the packet size is fixed, that is $T_{F}=T_{CF}$.

Hence, $R_{FTE}$ can be:

(20) $$R_{FTE}=\frac{T_{SL}}{T_{FSD}}=\frac{E(T_{SL})}{E(T_{FSD})}=\frac{T_{SL}}{E(T_{FSD})}$$

$R_{FTE}$ is determined by τ, *n* and $p_{c}$, if the size of the packet is fixed.

In [24], the collision probability $p_{c}$ is:

(21) $$p_{c}=1-{\left(1-\tau \right)}^{n-1}$$

**Figure 3:**
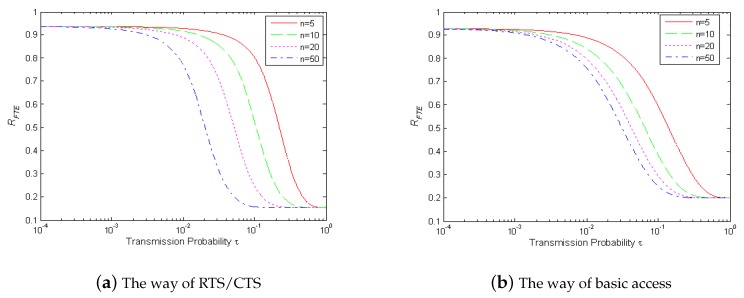
The relation of $R_{FTE}$, *τ* and *n*.

Thus, the frame transmission efficiency $R_{FTE}$ is only related with each nodes’ transmission probability τ, the number of neighbor nodes *n*, data length $T_{F}$ and collision data length $T_{CF}$. For simplicity, the data length is fixed to be 1000 bytes. The correlation of frame transmission efficiency, transmission probability τ and the number of neighbor nodes *n* is shown in Figure 3.

It is indicated from Figure 3 that the frame transmission efficiency decreases with the increasing of transmission probability. Especially, when τ is close to a certain threshold, if τ increases, the value of $R_{FTE}$ drops rapidly. It is also indicated from Figure 3 that increasing the number of neighbor nodes *n*, the value of $R_{FTE}$ drops when τ is certain.

The analysis above demonstrates that $R_{FTE}$ is sensitive to the channel congestion at the link layer. This conclusion motivated us to investigate a novel approach by using $R_{FTE}$ in congestion control in wireless ad hoc networks.

## 4. Protocol Design

Specifically, in the RECN scheme, when the value of $R_{FTE}$ is smaller than a given threshold, wireless routers will explicitly mark packets with a CE (Congestion Explicitness) bit in the IP (Internet Protocol) header. By this cross-layer ECN mechanism, the sender will adjust its sending rate on receiving congestion notification. RECN works at the MAC layer and uses the standard ECN mechanism. Thus, in the RECN scheme, there is only very small modification at the MAC layer. We note that RECN is a supporting design that is compatible with a wide range of TCP protocols.

The pseudo-code of RECN implementation is described as:
Mac802_11::check_pktTx(){// calculating total transmission time (including data packet, ACK delay and propagation delay)$T_{Trans}=txtime\left(pktTx\right)+txtime\left(ACK\right)+T_{prop}$;// recording the current time*bt* = now;$T_{SL}=T_{Trans}+T_{RTS}+T_{CTS}+3T_{SIFS};$$T_{FSD}=bt-st+T_{Trans}$; //*st* is the beginning time for transmitting.$R_{FTE}=\frac{T_{SL}}{T_{FSD}}$if $R_{FTE}<threshold$ // threshold is a given value.set CE bit to 1; // mark ECN bit}


Since the RTS/CTS mechanism is widely used in multi-hop wireless networks, we only take the RTS/CTS mechanism into consideration. However, our method is also suitable for the basic access mechanism.

## 5. Performance Evaluation

**Figure 4:**
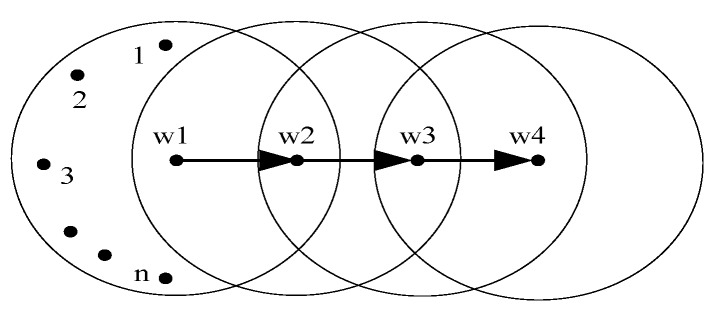
Simulation scenario.

In this section, we conduct simulations in NS2.27 (Network Simulator 2.27) [24,25]. The simulation scenario is shown in Figure 4. The transmission range of each node is 250 m, and the sensing range is 550 m. The route protocol is DSDV (Destination-Sequenced Distance-Vector routing protocol). We set the channel bandwidth (channel transmission rate) to 1 Mb and use 1000 bytes as the fixed size of each data packet. TCP data flows are sent by nodes (*1, ..., n*) from *w1* to *w4*, respectively. Simulations run for 300 s. We initiate UDP/constant bit rate (CBR) traffic flow as the background traffic starting from 0.5 s to 299.5 s at the speed of 500 kbps and initiate TCP/Constant Bit Rate (CBR) traffic flow starting from 1 s to 299 s at the speed of 800 kbps.

Table 1 gives the value of simulation scenario parameters.

We have made an experiment using UDP (User Datagram Protocol) flows as the background flows and calculated the number of RTS retransmission times, FSD, $R_{b}$ and $R_{FTE}$ of a TCP flow, as shown in Table 2.

Thus, in the following experiments, the threshold of RTS retransmission times is set to two; the threshold of FSD is set to 0.03; the threshold of $R_{b}$ is set to 0.75; and the threshold of $R_{FTE}$ is set to 0.6 based on the statistical results in Table 2.

### 5.1. Comparison with Other Metrics

We compare the throughput and packet loss rate of RECN with other metrics. We adopt RTS retransmission times [9], FSD [10], $R_{b}$ [13] and $R_{FTE}$ as the metrics of the indicator signal respectively, and also compare their performances with the case of no ECN.

**Figure 5:**
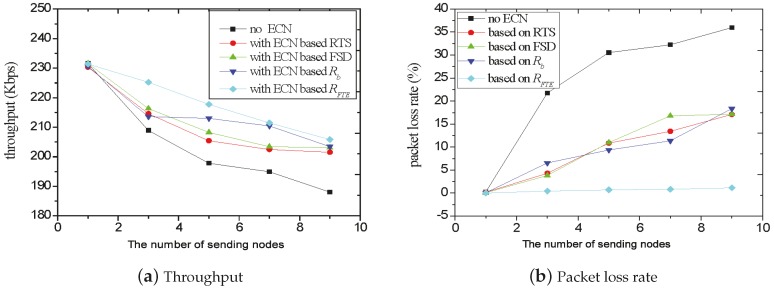
Comparison with other metrics.

In this section of the experiment, each sending node sent a TCP flow using UDP flows as the background flows. With the experiment, the number of sending nodes changed from one to 10. The results of throughput and packet loss rate are shown in Figure 5a,b, respectively.

Figure 5a shows that with the increasing number of sending nodes, the throughput decreases as a whole. However, compared with the no ECN situation, the decline of throughput is not so obvious as using ECN with the MAC layer’s metric. Specifically, the more sending nodes, the more obvious the improvement of the throughput is. This is because with more sending nodes, the possibility of congestion also increases. The sender using the ECN mechanism will reduce its sending rate when the congestion occurs.

In addition, Figure 5b tells us that the packet loss rate of no ECN is far greater than the others. For example, when number of flows is nine, the packet loss rate with no ECN is 0.35, with RTS, FSD and $R_{b}$ is 0.15 and with the $R_{FTE}$ we propose in this paper is only about 0.014.

To sum up, in the multi-hop ad hoc networks, the four metrics mentioned above can all improve network performance. However, the RECN metric we proposed can achieve the best improvement of both throughput and packet loss rate.

Thus, in the following sections, we focused on experiments to compare the performance of TCP with or without RECN.

### 5.2. TCP Throughput

Table 3 gives the throughput analysis when there are different numbers of TCP flows.

With the increased number of TCP flows, the total throughput sometimes tended to decline due to the increased channel competition. For example, while the number of TCP flows with RECN became four, the total throughput declined compared with three. When the number of TCP flows without ECN became five, the total throughput declined compared with four.

However, comparing with the no ECN situation, it is obvious from Table 3 that the total TCP throughput has an improvement by using RECN. For example, when the number of TCP flow is five, the total throughput increases by 95%.

**Figure 6:**
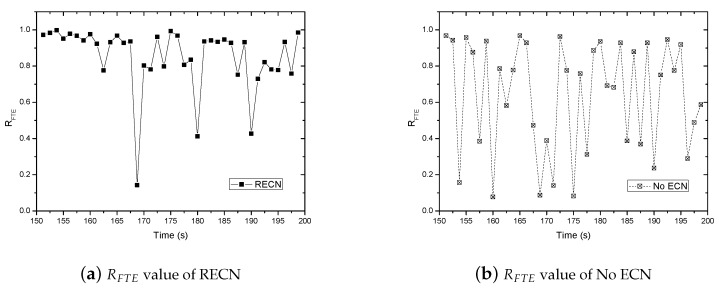
Comparison of the $R_{FTE}$ (TCP flow number: five).

Figure 6 gives the comparison of the instantaneous value of $R_{FTE}$ when there are five TCP connections.

It can be shown in Figure 6 that the average value of $R_{FTE}$ by using RECN is higher than that of the traditional TCP most of time. Combined with the statistics in Table 3, it can be concluded that the improvement of TCP throughput results from the improvement of data frame transmission efficiency.

### 5.3. Fairness Analysis

**Figure 7:**
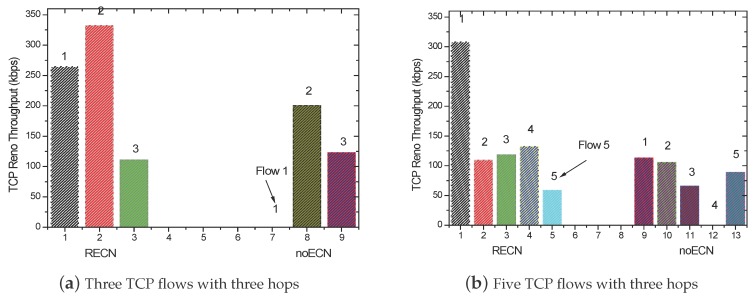
Comparison of fairness with three hops.

When the number of TCP connections is three and five, the throughput comparison of each flow is given by Figure 7a,b, respectively. With the traditional TCP, the throughput of one flow is close to zero. This is the inherent fairness problem of the IEEE 802.11 MAC protocol. However, by using RECN, TCP flows occupying the channel will slow down their sending rates when they received ACKs with congestion notification. Thus, these starved flows can get the chance to compete for the channel, and then, each flow can access the channel more fairly. Therefore, RECN is able to guarantee better fairness for each flow in multi-hop ad hoc networks.

**Figure 8:**
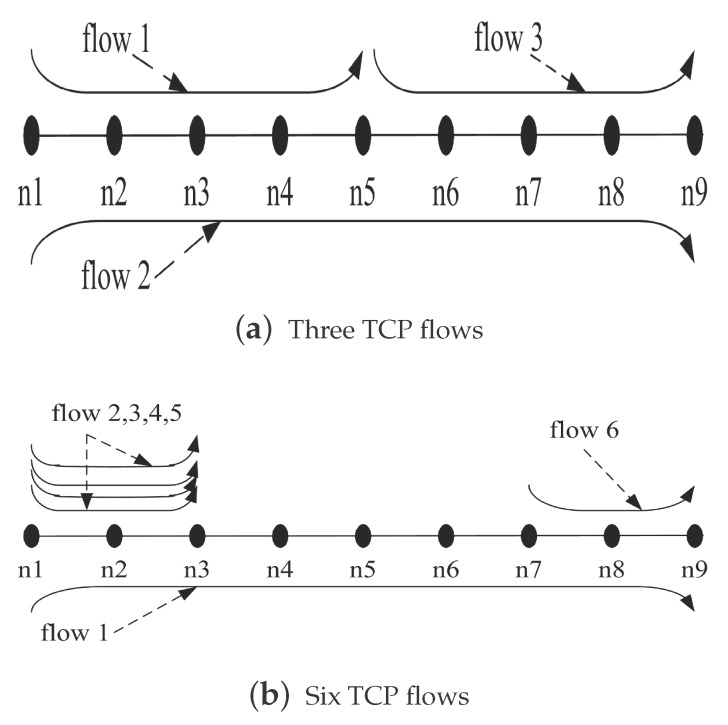
Simulation scenario with different hops.

**Figure 9:**
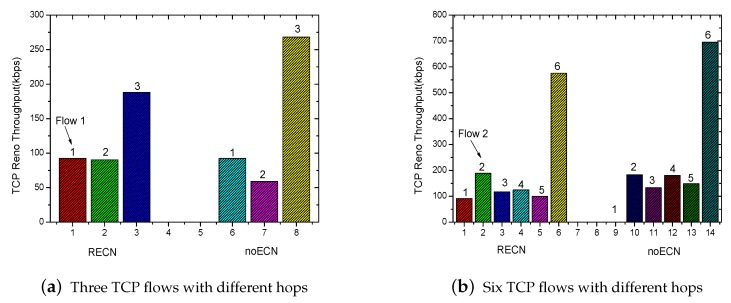
Comparison of fairness with different hops.

In addition, we also have tested the fairness among the TCP flows with different hops. As shown in Figure 8, we increase the number of nodes in the experiment scenario shown in Figure 4 to nine and then set the TCP flows with different hops. The throughput comparison of each flow is given by Figure 9a,b, respectively. With the traditional TCP, the starved flow still exists. By using RECN, each TCP flow with different hops has the chance to compete for the channel. The experiment shows again that RECN is able to guarantee better fairness for each flow in multi-hop ad hoc networks.

## 6. Conclusions

In wireless multi-hop ad hoc networks, congestion control plays an important role in reliable transmission. If the traditional TCP protocol is used, the network performance will degrade because of the poor interaction between the MAC and TCP layers. This paper proposes an MAC congestion notification scheme based on the frame transmission efficiency, which improves TCP performance with the congestion notification from the MAC layer. Analysis and simulation results show that our scheme outperforms traditional TCP in terms of throughput and fairness. Specially, it is realized with a small modification at the MAC layer, which can be applied to other existing TCP protocols. Therefore, the ECN mechanism has been widely used in recent years [26,27,28,29]. Thus, our scheme has good adaptability and generalization.

## Figures and Tables

**Table 1:** Simulation scenario parameter setting. prop refers to the propagation model. Mac refers to the MAC protocol. Ifq refers to interface queue. Ifqlen refers to the length of the interface queue. SIFS refers to the Short Interframe Space. The Phy header refers to the length of the Physical layer header. The Mac header refers to the length of the media access layer header.

Parameters	Setting
prop	TwoRayGround
mac	802.11
ifq	Drop Tail/PriQueue
ifqlen	20
SIFS	10 μs
Phy header	192 bits
Mac header	224 bits
RTS	160 bits + Phy header
CTS, ACK	112 bits + Phy header

**Table 2:** The value of RTS retransmission times, Frame Service Delay (FSD), $R_{b}$ and $R_{FTE}$.

The Number of Background Flows (UDP Flows)	1	2	3	4
RTS retransmission times	1.349	1.688	2.068	2.109
FSD (ms)	20.234	21.289	23.560	24.395
$R_{b}$	0.66237	0.69409	0.71038	0.72191
$R_{FTE}$	0.58043	0.57561	0.57334	0.57322

**Table 3:** TCP throughput analysis. Compare the two situation: RECN and No ECN.

TCP Flow Number	Total Throughput *(kbps)*		Average Value of $R_{FTE}$	
	RECN	No ECN	RECN	No ECN
1	244.58	232.39	0.761	0.645
2	556.11	317.64	0.760	0.640
3	707.89	323.81	0.758	0.646
4	640.80	399.18	0.756	0.613
5	727.27	373.71	0.753	0.624
